# Safety and efficacy of vacuum bottle plus catheter for drainage of iatrogenic pneumothorax

**DOI:** 10.1186/s12890-022-02009-8

**Published:** 2022-06-07

**Authors:** Shih-Yu Chen, Yao-Wen Kuo, Chao-Chi Ho, Huey-Dong Wu, Hao-Chien Wang

**Affiliations:** 1 Department of Internal Medicine, National Taiwan University Hospital Hsin-Chu Branch, Hsinchu, Taiwan; 2 Department of Integrated Diagnostic and Therapeutics, National Taiwan University Hospital, No. 7, Chung-Shan South Road, Taipei, 10002 Taiwan; 3 Division of Pulmonary Medicine, Department of Internal Medicine, National Taiwan University Hospital, Taipei, Taiwan; 4 Department of Medicine, National Taiwan University Cancer Center, Taipei, Taiwan

**Keywords:** Drainage of pneumothorax, Iatrogenic pneumothorax, Intrapleural pressure, Vacuum bottle

## Abstract

**Background:**

Iatrogenic pneumothorax is common after thoracic procedures. For patients with pneumothorax larger than 15%, simple aspiration is suggested. Although vacuum bottle plus non-tunneled catheter drainage has been performed in many institutions, its safety and efficacy remain to be assessed.

**Methods:**

Through this prospective cohort study (NCT03724721), we evaluated the safety and efficacy of vacuum bottle plus non-tunneled catheter drainage. Patients older than 20 years old who developed post-procedural pneumothorax were enrolled. A non-tunneled catheter was placed at the intersection of the midclavicular line and the second intercostal space. A 3-way stopcock, a drainage set, and a digital pressure gauge were connected. The stopcock was manipulated to connect the pleural space to the pressure gauge for measurement of end-expiration intrapleural pressure or to the vacuum bottle for air drainage. The rate of successful drainage, the end-expiration intrapleural pressure before, during, and after the procedure and the duration of hospitalization were recorded.

**Results:**

From August 2018 to February 2020, 21 patients underwent vacuum bottle plus catheter drainage (intervention group) and 31 patients received conservative treatment (control group). The end-expiration intrapleural pressure of all patients remained less than − 20 cmH_2_O during drainage. No procedure related complication was observed. Large pneumothorax (≥ 15%) was associated with higher risk of persistent air leak (Odds ratio 12, 95% CI 1.2–569.7). Vacuum bottle assisted air drainage yielded shorter event-free duration than that of conservative treatment (2 days vs 5 days [interquartile range 1–4 days vs 3–7 days], *p* < .05). Vacuum bottle assisted air drainage also help identifying patients with persistent pneumothorax and necessitate the subsequent management. The event-free duration of persistent air leak in the intervention group was also comparable with that of conservative treatment (5 days vs 5 days [interquartile range 5–8 days vs 3–7 days], *p* = .45).

**Conclusions:**

Vacuum bottle plus catheter drainage of iatrogenic pneumothorax is a safe and efficient procedure. It may be considered as an alternative management of stable post-procedural pneumothorax with size larger than 15%.

*Trial registration* The study protocol was approved by the Research Ethics Committee of National Taiwan University Hospital (No. 201805105DINA) on 6th August, 2018. The first participant was enrolled on 23rd August, 2018 after Research Ethics Committee approval. This clinical trial complete registration at U.S. National Library of Medicine clinicaltrials.gov with identifier NCT03724721 and URL: https://clinicaltrials.gov/ct2/show/NCT03724721 on 30th October, 2018.

**Supplementary Information:**

The online version contains supplementary material available at 10.1186/s12890-022-02009-8.

## Introduction

Iatrogenic pneumothorax is a common complication resulting from many pulmonary interventions. The reported incidence of iatrogenic pneumothorax is as follows: 0.8–1.4% in patients undergoing radial endobronchial ultrasound-guided transbronchial lung biopsy, approximately 2–6% among patients undergoing thoracentesis, 0.6–9% in patients undergoing echo-guided biopsy, and as high as 38% among patients undergoing CT (computed tomography)-guided lung biopsy [1–8]. The British Thoracic Society guidelines in 2010 suggested that observation alone is adequate for majority of the cases of iatrogenic pneumothorax and that, if intervention is required, simple aspiration be considered [9]. Several studies have reported that catheter aspiration for iatrogenic pneumothorax is cost effective and with few complications [10–12].

The introduction of thoracentesis to remove either fluid or air through vacuum bottle drainage system can be traced back to the 1950s and it had been widely adopted in many clinical settings [13, 14]. Compared with manual aspiration, vacuum bottle assisted thoracentesis requires less time and allows uninterrupted drainage without repeatedly manipulating 3-way stopcock. Although this procedure had long been adopted by many medical facilities in Taiwan, it has rarely been described in the literature, especially data regarding its safety and efficacy. To the best of our knowledge, only one pilot study documented the complication rate of vacuum bottle fluid drainage, which was 9.8% [14]. However, the study was conducted at a single center where vacuum bottle drainage was not routinely employed. In addition, the flow rate of fluid drainage, a key component when applying negative pressure drainage system, was not reported in the study. Further, data on air removal by vacuum bottle drainage system were lacking. Thus, through the current study, we aim to investigate the safety and efficacy of this procedure to provide an evidence-based approach for this routine clinical practice.

## Methods

### Study design and data collection

This prospective interventional study was conducted in the general chest ward in National Taiwan University Hospital, a tertiary center in northern Taiwan, from August 2018 to February 2020. We enrolled adult patients who developed iatrogenic pneumothorax after undergoing invasive thoracic procedures.

The inclusion criteria were patients with radiographic evidence of pleural line after lung biopsy (echo-guided, bronchoscopic, or CT-guided biopsy) and whose size of pneumothorax was more than 15%, measured by Rhea’s criteria [15]. The exclusion criteria were age younger than 20 years, bleeding tendency, and hemodynamic instability. Patients fulfilling the above criteria and agreed to participate were prospectively enrolled for the safety analysis.

In this institute, patients with size of iatrogenic pneumothorax less than 15% would receive conservative treatment, namely oxygenation therapy and image monitoring first. If the size of pneumothorax enlarged or hemodynamic instability occurred, further intervention would be proceeded. The approach was based on BTS guideline which reported that up to 80% of pneumothoraces smaller than 15% have no persistent air leak [9]. Besides, there appears to be more technical difficulty for physicians to aspirate smaller size pneumothorax. These subjects were thus retrospectively evaluated for the efficacy analysis out of ethical concern. All patients were admitted before biopsy and hospitalized for post biopsy care such as monitoring vital signs, severity of pain, degree of dyspnea or hypoxia, presence of hemoptysis and given immediate symptomatic treatment accordingly.

The study protocol was approved by the Research Ethics Committee of National Taiwan University Hospital (No. 201805105DINA). This clinical trial was registered at clinicaltrials.gov with identifier NCT03724721 on 30/10/2018. Written informed consent was obtained from each subject before enrolment. The whole research process was performed in accordance with the Declaration of Helsinki.

### Study protocol

**Fig. 1:**
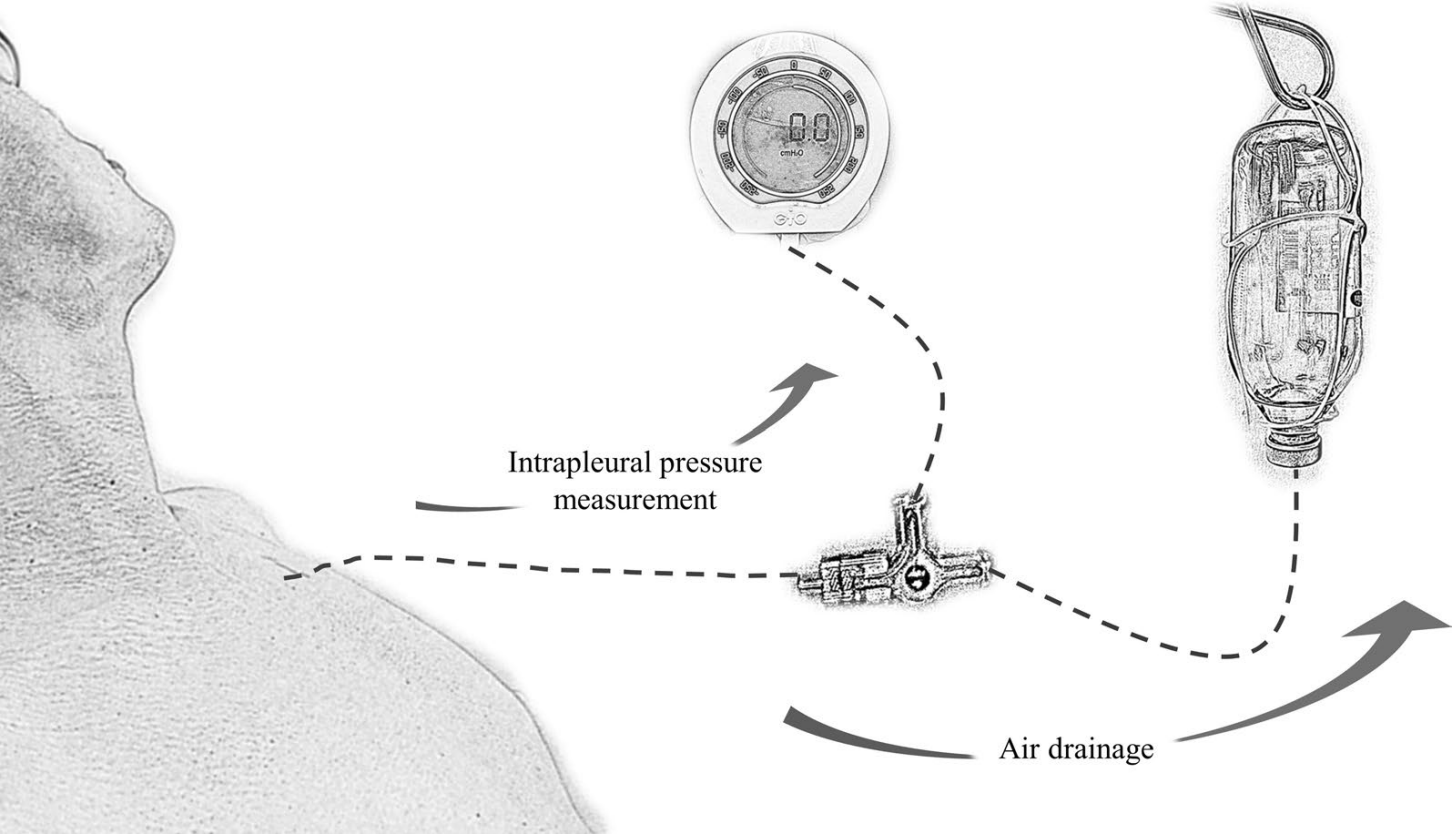
A schematic diagram of how vacuum bottle plus non-tunneled catheter air drainage and end-expiration intrapleural pressure were measured

The standardized procedure for drainage of pneumothorax by vacuum bottle is described in the Fig. 1 and next section. To avoid rapid intrapleural pressure change and suction trauma, we made sure the flow rate of air drainage was slow by keeping the air bubble in one straight line throughout the procedure (Additional file 1: Video S1).

**Fig. 2:**
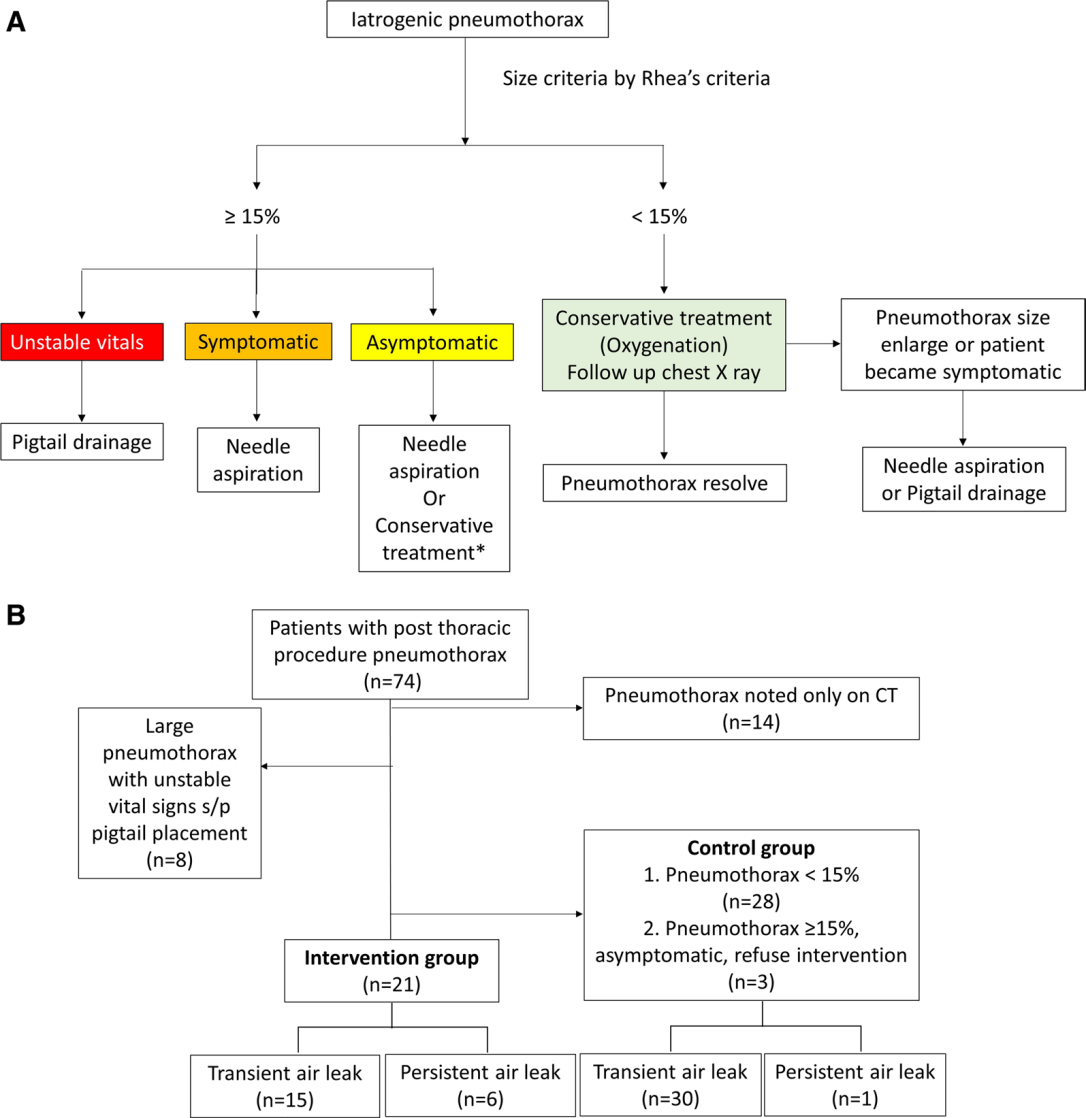
**A** Initial treatment algorithm of iatrogenic pneumothorax. Needle aspiration: vacuum bottle plus catheter drainage. *Decision was made by shared decision making. Conservative treatment: oxygenation. **B** Flow chart of subject recruitment process for this study. *Transient air leak denotes no further enlargement of pneumothorax after vacuum bottle assisted aspiration or oxygenation only; Persistent air leak represents that pneumothorax enlarged either after initial management or need further intervention

The decision making was based on the treatment algorithm in Fig. 2A. The flow chart of enrollment was presented in Fig. 2B. Patients with either smaller pneumothorax only visible on CT or large pneumothorax and unstable vital signs were excluded. Patients with pneumothorax size smaller than 15% and stable vital signs received oxygenation only and were closely monitored by image once daily. In participants with pneumothorax equal to or larger than 15%, symptomatic participants are recommended to have vacuum bottle assisted aspiration for symptom relive while asymptomatic patients would have shared decision making before intervention.

### Procedure protocol

After obtaining inform consent, the patient was placed in supine position with head of bed at an angle of 30°–45°. We injected 20 ml normal saline into the vacuum bottle (Sterile bottle 500 ml; Taiwan Biotech Co., Taoyuan, Taiwan) for visualization of air bubbles. The vacuum bottle was then turned upside down and hung up on a drip stand at the same level of the patient’s chest, and connected to a drainage set (Disposable infusion set; Perfect Medical Industry Co., Ho Chi Minh, Vietnam). We used chest ultrasound to reassure the presence of pneumothorax below the catheter insertion site, located at the second intercostal space, by absence of sliding sign and presence of Barcode sign or Stratosphere sign. The skin was sterile with chlorhexidine-based solution and allowed to dry. After local anesthesia, we inserted the over-the-needle catheter (Surflo®; Terumo Medical Co., Laguna, Philippines) into pleural space, removed the needle, and attached the catheter to three-way stopcock (Discofix®; B. Braun, Melsungen, Germany). We connected the other two ends of the three-way stopcock to the drainage set and the digital pressure gauge (GiO 6, JITTO; GaleMed Co., Ilan, Taiwan). The three-way stopcock was manipulated to connect the pleural space to the digital pressure gauge to check intrapleural pressure at end-expiration. We then manipulated the three-way stopcock again to connect the pleural space to the drainage set to start to drain the pneumothorax with formation of air bubble in one straight line (Additional file 1: Video S1). We manipulated the three-way stopcock and checked intrapleural pressure at end-expiration every 30 s. After no more air could be drained, we checked intrapleural pressure again and then remove the catheter. Chest X-rays were checked immediately after the procedure and on the next day (Additional file 2: Text S1). If pneumothorax enlarged in size ≥ 15% in the following chest X-ray, persistent air leakage is considered and a rescue 8 Fr. pigtail (Hydrophilic drainage catheter; Bioteque Co., Taipei, Taiwan) would be placed with water seal system. Daily chest X ray would be arranged until pigtail could be removed.

### Data collection and outcome measure

In the intervention group, the subjects’ baseline characteristics and study parameters were recorded prospectively, including age; gender; underlying disease; chest X-ray before, immediately after, and on the next day after the drainage; thoracic procedures (thoracentesis, echo-guided biopsy, bronchoscopic biopsy, CT-guided biopsy); and the size of the pneumothorax, as defined by the Rhea’s criteria. The primary outcomes included the rate of successful drainage with the definition being persistent full lung expansion or partial lung expansion with size less than 15% by the Rhea’s criteria immediately after, and on the next day of the procedure; and the end-expiration intrapleural pressure before, at 30-s intervals during, and at the end of the procedure. The secondary outcomes included the patient’s discomfort recorded in the numeric rating scale from 0 to 10 before, during and after the procedure; and any possible side effects, such as re-expansion pulmonary edema, hemothorax, and subsequent tension pneumothorax.

In the control group, the baseline demographics were retrospectively collected and recorded in the same way as the intervention group. In this center, patients who were receiving oxygenation only treatment for pneumothorax underwent chest X-ray every day and were not allowed to be discharged until the chest X-ray showed near total resolution of pneumothorax. Because of prolonged admission that occurred often owning to the requirement of extra hospitalization such as waiting for other diagnostic studies or treatments for underlying diseases, the event-free date was defined as the date when the primary care team shifted the problem of post-biopsy pneumothorax from the list of active problems to inactive ones.

A secondary analysis was arranged for further outcome assessment. Patients in the intervention group were divided into “transient air leak” group and “persistent air leak” group. Transient air leak was defined by successful air drainage with vacuum bottle assisted air drainage once only. Persistent air leak was defined by continued enlargement of pneumothorax in the subsequent chest X-ray follow up that needed further management such as pigtail placement. In order to compare patients with similar sized pneumothorax with the intervention group, a 10% cutting point was made in the control group. A comparison of the event-free durations was made between intervention subgroups and patients with pneumothorax ≥ 10% in the control group.

### Statistical analysis

Baseline characteristics are expressed as median with interquartile range (IQR), mean with standard deviation (SD), or number with proportion, as appropriate. Continuous variables were compared using Wilcoxon rank-sum test or Wilcoxon signed-rank test, as appropriate. Categorical data were compared using the Chi-square test or Fisher’s exact test. A *P* < 0.05 was considered statistically significant. All statistical analyses were performed using STATA version 14 software (StataCorp LLC, College Station, TX, USA).

## Results

### Characteristics of the study cohort

A total of 74 patients were screened, and 21 patients who met the inclusion criteria and provided informed consent were enrolled as the intervention group. Another 31 patients received oxygenation only and enrolled as control group. Among them, 28 patients had radiographically obvious pneumothorax but pneumothorax size less than 15% by Rhea’s criteria and 3 patients had asymptomatic pneumothorax larger than 15% but refused intervention after shared decision making (Fig. 2). The baseline demographics of all subjects are summarized in Table 1. The median age of the subjects was 66.5 (IQR 61–70) years, and 27 of them were male. Among the 52 iatrogenic pneumothorax events, CT-guided biopsy accounted for 40 events. Only one patient had a previous experience of pneumothorax, which was also iatrogenic. In total, 41 patients had lung cancer and 16 of them underwent rebiopsy for progressive disease. The baseline characteristics between the intervention group and control group were similar, except that patients in the control group were heavier than those in the intervention group (58.4 kg vs 63.1 kg, *P* = 0.043). However, the body mass index (BMI) did not statistically differ between the two groups (22.4 vs 24.6 kg/m^2^, *P* = 0.061).

**Table 1:** Baseline demographics of the study subjects

	Total	Intervention group	Control group	*p* value
	(n = 52)	(n = 21)	(n = 31)	
Age, year	66.5(61,70)	64(62,69)	68(59,73)	0.582
Male	27(51.9%)	10(47.6%)	17(54.8%)	0.609
Height, cm	160(152.8,166.8)	158.3(152.6,164.7)	162(152.8,167.3)	0.244
Weight, kg	61.5(55.2,68.7)	58.4(47.6,64.8)	63.1(57.1,71.6)	0.043
BMI	23.3(21.1,26.3)	22.4(20.6,23.7)	24.6(21.4,27.0)	0.061
Current smoker	18(34.6%)	7(33.3%)	11(35.5%)	1
Procedure				
CT-guided biopsy	40	14	26	0.518
Echo guided biopsy	2	1	1	
Bronchoscopic biopsy	4	2	2	
Thoracentesis	6	4	2	
Underlying disease				
Lung cancer	41	17	24	0.501
COPD	8	2	6	
Asthma	3	0	3	
Bronchiectasis	1	0	1	
Previous pneumothorax	1	1	0	

Data are presented as n (%) or median (interquartile range)

COPD, chronic obstructive pulmonary disease; control group, oxygenation only group

### Safety analysis

All 21 patients in the intervention group completed vacuum bottle assisted air drainage smoothly. The end-expiratory intrapleural pressure of all patients remained less than − 20 cmH_2_O during drainage (Additional file 2: Fig. S1). The median time of air removal was 90 (IQR 60–180) seconds. The median numeric rating scale for discomfort scores out of 10 before, during (30 s after initiation), and after the procedure in patients in the intervention group who were receiving vacuum bottle assisted drainage were 1, 1, and 0, respectively (Additional file 2: Fig. S2). No procedure-related bleeding, infection, re-expansion pulmonary edema, or mortality was observed.

### Efficacy and subgroup analysis

The median size of pneumothoraces were 19.6% (IQR 16–24%) and 8.8% (IQR 6.2–13.2%) by Rhea’s criteria in the intervention and control group, respectively. Table 2 showed that there were more proportion of patients with persistent air leakage in large (≥ 15%) and/or symptomatic pneumothoraces as compared to small (< 15%) or asymptomatic pneumothoraces. The odds ratio for persistent air leakage in large and/or symptomatic pneumothoraces as compared to small or asymptomatic pneumothorax was 12 (95% confidence interval 1.2–569.7, *p* < 0.05).

**Table 2:** Patient outcomes

	Large and/or symptomatic pneumothorax	Small or asymptomatic large pneumothorax	*p* value
	(n = 21)	(n = 31)	
Pneumothorax size (%)	19.6 (16,24)	8.8 (6.2,13.2)	< 0.05
Outcome			
Transient air leak	15 (71.4%)	30 (96.8%)	0.013
Persistent air leak	6 (28.6%)	1 (3.2%)	

Data are presented as n (%) or median (interquartile range)

Large pneumothoraces are defined by size ≥ 15% by Rhea’s criteria

Figure S3 (Additional file 2: Fig. S3) showed the recovering course of patients with pneumothoraces who received conservative treatment only. There was a moderate-to-high positive correlation between pneumothorax size and length of event-free duration, r = 0.72, *p* < 0.05. There was a dramatic increase of event-free duration with an intercept at size of 10%. The median length of event-free duration was 1 day (IQR 1–1 day) and 5 (IQR 3–7) days in smaller size pneumothorax (< 10%) and larger size pneumothorax (≥ 10%), respectively, *p* < 0.05.

**Fig. 3:**
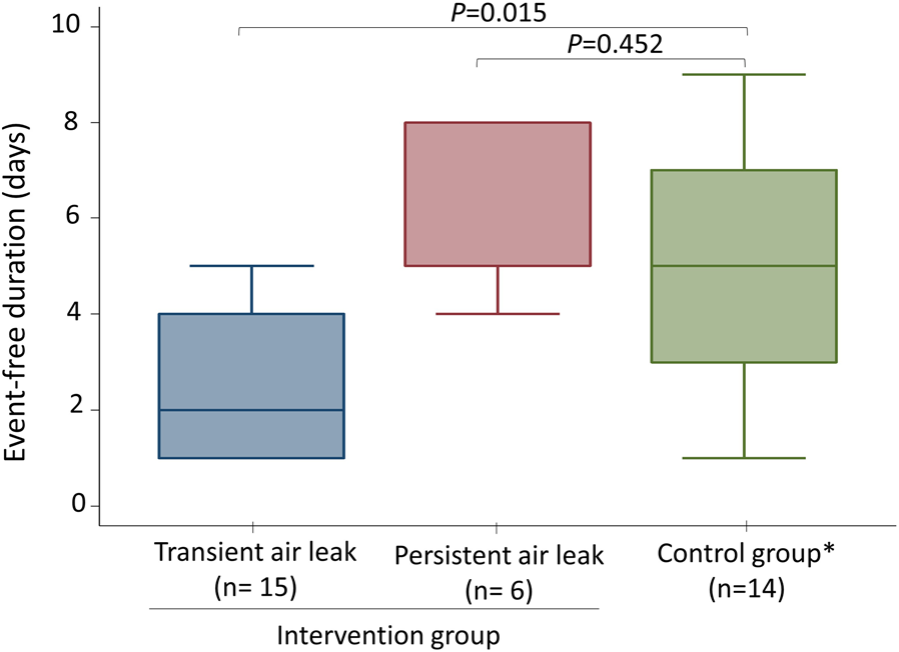
Box plots for comparison of time to event-free duration between the intervention group and control group. Median (interquartile range); n = patient number. *Patients with comparable size of pneumothorax (≥ 10%) with the intervention group were included for comparison

In the intervention group, all patients with transient air leak recovered by vacuum bottle assisted air drainage. The median length of event-free duration was significantly less than patients with similar pneumothorax size in the control group, 2 (IQR 1–4) days versus 5 (IQR 3–7) days, *p* < 0.05 (Fig. 3). All patients with persistent air leak were documented soon after vacuum bottle assisted air drainage and the pneumothoraces resolved totally by subsequent small bore pigtail placement. The median length of event-free duration was comparable with similar size pneumothorax in the control group, 5 (IQR 5–8) days versus 5 (IQR 3–7) days, *p* = 0.45 (Fig. 3).

## Discussion

Through this prospective study, we reported that the vacuum bottle plus non-tunneled catheter drainage of pneumothorax is a safe and efficacious procedure. In addition, we provide a detailed clinical course concerning oxygenation only with watchful waiting and intervention, which may aid decision-making regarding post procedural pneumothorax.

Simple aspiration of air or fluid through catheter plus vacuum bottle has been performed in many hospitals in Taiwan. Compared with manual drainage, a vacuum bottle-assisted drainage facilitates continuous removal of air or fluids, diminishing the necessity of repeated 3-way stopcock and syringe manipulation which could be annoying and time-consuming during large-volume thoracentesis [13, 16]. Yamagami et al*.* evaluated 72 post-CT guided biopsy pneumothoraxes that required needle aspiration; the mean volume of air was 527 mL, with the largest volume being 2700 mL. In a similar study conducted by Faruqui et al*.*, the mean aspirated air volume was 680 (200–2000) mL. A 50-mL syringe would require approximately 10–50 repeated manipulations, which would be laborious and time-consuming [12, 17].

However, the possibility of large negative pressure exerted by the vacuum bottle on the pleural space makes the safety of this procedure a concern [14]. A rapid re-expansion of the collapse lung may lead to so called re-expansion pulmonary edema. Although rare, the related mortality might be as high as 20% [18]. The main factors contributing to re-expansion pulmonary edema include collapse of the lung for more than 3 days, use of negative intrapleural pressure to rapidly re-expand the collapse lung, and the removal of more than 1 L of effusion [19, 20]. Feller-Kopman et al*.* suggested that large-volume thoracentesis is feasible as long as the patient is symptom-free or the end-expiration intrapleural pressure is maintained below − 20 cmH_2_O [19]. In this study, the time from the development of pneumothorax to intervention were all within hours. Besides, the air drainage flow rate was slow by keeping the formation of air bubble in one straight line throughout the procedure. Lastly, by intermittently monitoring intrapleural pressure during negative pressure drainage, we proved that the vacuum bottle-assisted drainage is safe, and the end-expiratory intrapleural pressure remained less than 20 cmH_2_O throughout the procedure (Additional file 2: Fig. S1) [21].

In the current cohort study, 15 out of 21 (71.4%) patients achieved lung expansion by vacuum bottle plus non-tunneled catheter drainage, with the remaining 6 (28.6%) patients requiring pigtail catheter drainage as a rescue. This finding is comparable with previous studies, which reported a lung expansion rate of approximately 57.1–94.1% by manual needle aspiration [1, 9–12, 17]. The median event-free period in this study was 2 (IQR 1–4) days in the vacuum bottle plus non-tunneled catheter drainage only group. This finding is compatible with previous studies, wherein the median hospital duration ranged from 1 to 5 days (Additional file 2: Table S1) [10–12, 17, 22]. The event-free duration was almost equal to that in the subgroup of patients in the oxygen only group with size of pneumothorax less than 10% and shorter than that in patients with larger size of pneumothorax in this cohort. Moreover, all patients who required subsequent pigtail placement achieved full lung expansion in this study, with an event-free period of 5 (IQR 5–8) days, which was comparable to the event-free duration of 6–7 days with primary tube thoracostomy reported in previous studies [10, 12].

In this study, discomfort measurement before, during, and after the procedure was recorded as a very low score. Most patients reported no increased procedure related discomfort, except for minimal pain while receiving the local anesthesia injection before catheter placement. A similar result was reported by Faruqi et al., and the mean pain scores for simple aspiration and intercostal tube drainage were 1.6 and 4.0 respectively [17]. Moreover, the lack of indwelling catheter not only alleviated the irritable sensation that occurred owning to the presence of a foreign body object inside the body, which enabled patients to mobilize freely without limitation or fear of tube dislodgement, but also reduced the clinical burden of tube care on the nursing stuff and the medical costs [23]. Finally, no complication occurred in patients who underwent vacuum bottle plus non-tunneled catheter drainage in this study. Hence, we can conclude that vacuum bottle plus non-tunneled catheter drainage, similar to previously reported manual simple air aspiration, is a safe and efficient procedure (Additional file 2: Table S1) [10–12, 17, 22].

Unlike other previous studies, we also evaluated the clinical outcomes of the patients in the oxygenation only group and found that the event-free duration differed dramatically with the size of pneumothorax, considering 10% as the cut-off point (Additional file 2: Fig. S3). We also found that patients with transient air leak had shorter event-free duration after vacuum assisted air drainage as compared to control group with comparable size of pneumothorax. In addition, vacuum assisted air drainage aid in identifying persistent air leak. After subsequent management, the event-free duration was similar to control group (Fig. 3). Thus, we suggest that initial management may entail simple aspiration by a chest specialist, because it may not only reduce the duration of hospital stay and medical costs but also help determine the next treatment action, especially in patients with pneumothorax size larger than 15%.

This study has several limitations. First, we did not perform a head-to-head comparison between catheter plus vacuum bottle-assisted air drainage and manual needle aspiration. Besides, a retrospective comparison may preclude validation of the data presented. A direct prospective randomized controlled trial may be required to overcome the limitation in the future. Second, this study was conducted in a single center and with predominantly CT-guided biopsy induced pneumothorax, which may prevent generalization of the findings. Third, there is no universal method for pneumothorax size measurement. In addition, all these measurements are based on chest radiography, a 2-dimensional imaging modality, which may show disagreement on size of pneumothorax from each other. Further, the pleural line did not appear smooth in most patients, which could render the calculation incorrect for formulas that use only one parameter, such as Light’s formula or the interpleural distance at the hilum level considered by the British Thorax Society. This study adopted Rhea’s criteria, as we consider 3 sites of distance measurement for calculation, which suits most cases of lung collapse with varying shapes of pleural line. More accurate evaluation methods such as CT imaging may be considered and a universal consensus may be required, especially in asymptomatic patients with subjective large pneumothorax. Finally, the results of the current study can be applied only in patients with stable vital signs and not in those receiving positive mechanical ventilation. It may not be applied to patients with idiopathic pulmonary fibrosis or other interstitial lung diseases as the course of pneumothorax may be different in such underlying conditions [24].

## Conclusion

Through this prospective cohort study, we proved that vacuum bottle plus non-tunneled catheter air drainage is a safe and efficacious air drainage method. It is associated with a short hospital stay, less patient discomfort, and reduced medical costs; moreover, this procedure could help determine the necessity of further management. It may be considered as an alternative option in stable patients with symptomatic post-procedural pneumothorax or pneumothorax size larger than 15%, as measured by Rhea’s criteria.

## Supplementary Information

**Additional file 1: Video S1.** A video demonstrating the process of air drainage. To avoid rapid intrapleural pressure change and suction trauma, the flow rate of air drainage was slow and air bubbles were kept in one straight line throughout the procedure.

**Additional file 2: Text S1.** Procedure protocol with illustrating figures. **Fig. S1.** Pressure-time curve of end-expiratory intrapleural pressure measurement during air drainage by vacuum bottle plus non-tunneled catheter in 21 patients. **Fig. S2.** Box plots in combination with scattered dot plots for participants' discomfort in the numeric rating scale of 10 recording before, during and after the procedure.  **Fig. S3.** Scatter plots of time to event-free duration to pneumothorax size in control group. **Table S1.** Comparison of studies employing simple air aspiration in patients with iatrogenic or traumatic pneumothoraces.

## Data Availability

All data generated or analyzed during this study are included in this published article and its supplementary information files.

## Abbreviations

CT: Computed tomography
COPD: Chronic obstructive pulmonary disease
EIP: End-expiratory intrapleural pressure
Fr.: French
IQR: Interquartile range
SD: Standard deviation
