# Supplementary material for: Safety and efficacy of vacuum bottle plus catheter for drainage of iatrogenic pneumothorax
Source: BMC Pulm Med. 2022 Jun 7;22:221. doi: 10.1186/s12890-022-02009-8 (PMC9175504; doi:10.1186/s12890-022-02009-8)
Supplement: Supplementary file 2 — Additional file 2: Text S1. Procedure protocol with illustrating figures. Fig. S1. Pressure-time curve of end-expiratory intrapleural pressure measurement during air drainage by vacuum bottle plus non-tunneled catheter in 21 patients. Fig. S2. Box plots in combination with scattered dot plots for participants' discomfort in the numeric rating scale of 10 recording before, during and after the procedure. Fig. S3. Scatter plots of time to event-free duration to pneumothorax size in control group. Table S1. Comparison of studies employing simple air aspiration in patients with iatrogenic or traumatic pneumothoraces. [file 12890_2022_2009_MOESM2_ESM.docx]

**Supplementary Text S1.** Procedure protocol with illustrating figures.


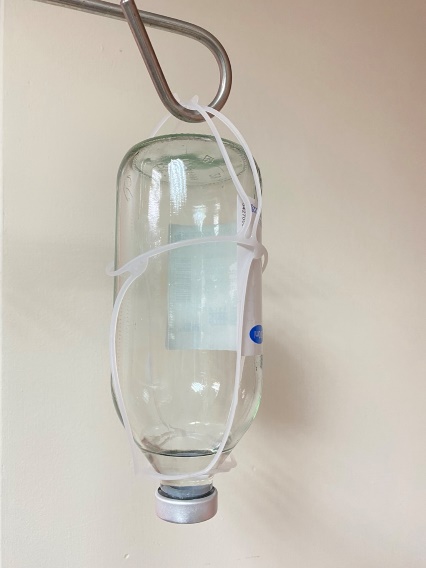
1. Preparations: (1) Obtain Inform consent, (2) Position the patient’s head of bed at an angle of 30 to 45 degrees, (3) Injected 20ml normal saline into the vacuum bottle for visualization of air bubbles, (4) Turn the vacuum bottle upside down and hang up on a drip stand at the same level of the patient’s chest, and connect to drainage set, (5) Identify the catheter insertion site with chest ultrasound by absence of sliding sign and presence of Barcode sign or Stratosphere sign, lung point, (6) Sterile the skin with chlorhexidine-based solution and allow to dry.

Vacuum bottle filled with 20ml normal saline hung upside down


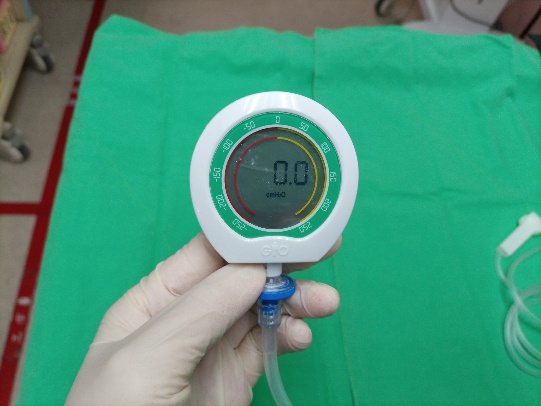

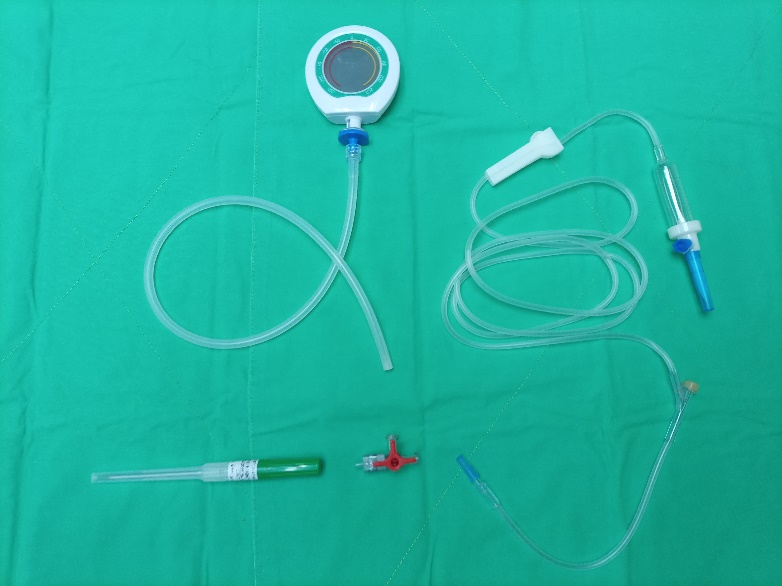
2. Procedure: (1) Local anesthesia, (2) Insert the over-the-needle catheter into pleural space, remove the needle, and attach the catheter to three-way stopcock, (3) Connect the other two ends of the three-way stopcock to the drainage set and the digital pressure gauge (GiO 6, JITTO), (4) Manipulate the three-way stopcock, connecting the pleural space to the digital pressure gauge, check intrapleural pressure at end-expiration, (5) Manipulate the three-way stopcock, connecting the pleural space to the vacuum bottle, then manipulate the drainage set to start to drain the pneumothorax with formation of air bubble in one straight line. (Additional file 1: Video S1) (6) Slowly elevate the patient’s head of bed to a 90-degree angle, (7) Manipulate the three-way stopcock and check intrapleural pressure at end-expiration 1 min later, (8) Connect to second vacuum bottle if indicated, (9) If no more air was drained, check intrapleural pressure again, then remove the catheter, (10) Follow up chest X-ray immediately after the procedure and on the next day.

Intrapleural pressure measurement at end-expiration

A schematic diagram for the connection of the non-tunneled catheter, 3-way stopcock, digital pressure gauge, and the drainage set

**Supplementary Figure S1.** Pressure-time curve of end-expiratory intrapleural pressure measurement during air drainage by vacuum bottle plus non-tunneled catheter in 21 patients.


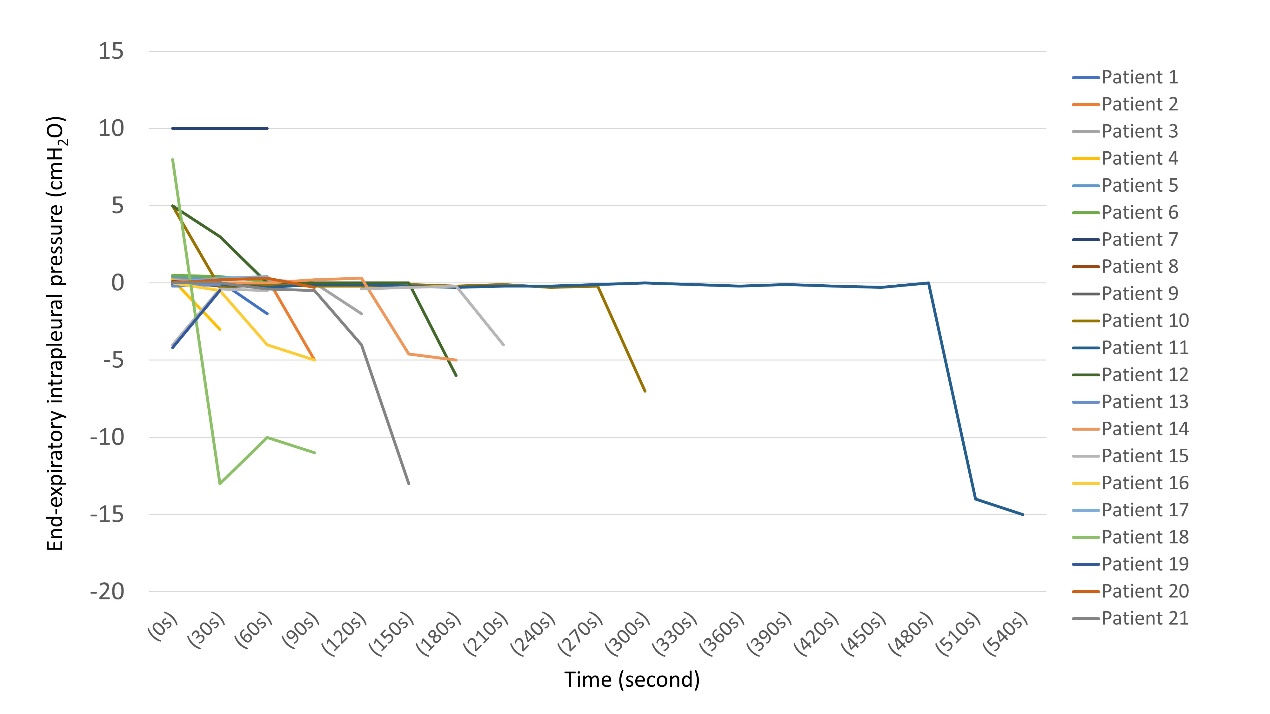


**Supplementary Figure S2.** Box plots in combination with scattered dot plots for participants’ discomfort in the numeric rating scale of 10 recording before, during and after the procedure.


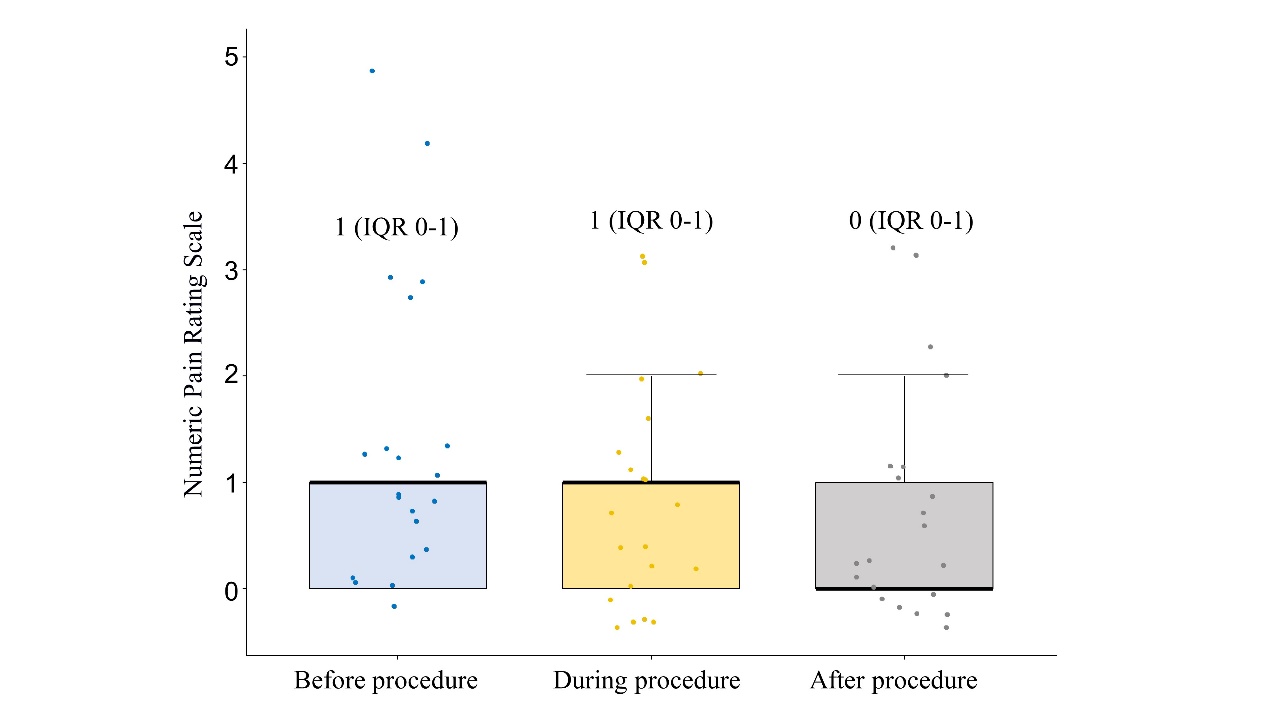


Median (interquartile range)

**Supplementary Figure S3.** Scatter plots of Time to Event-free Duration to Pneumothorax Size in Control group.

**
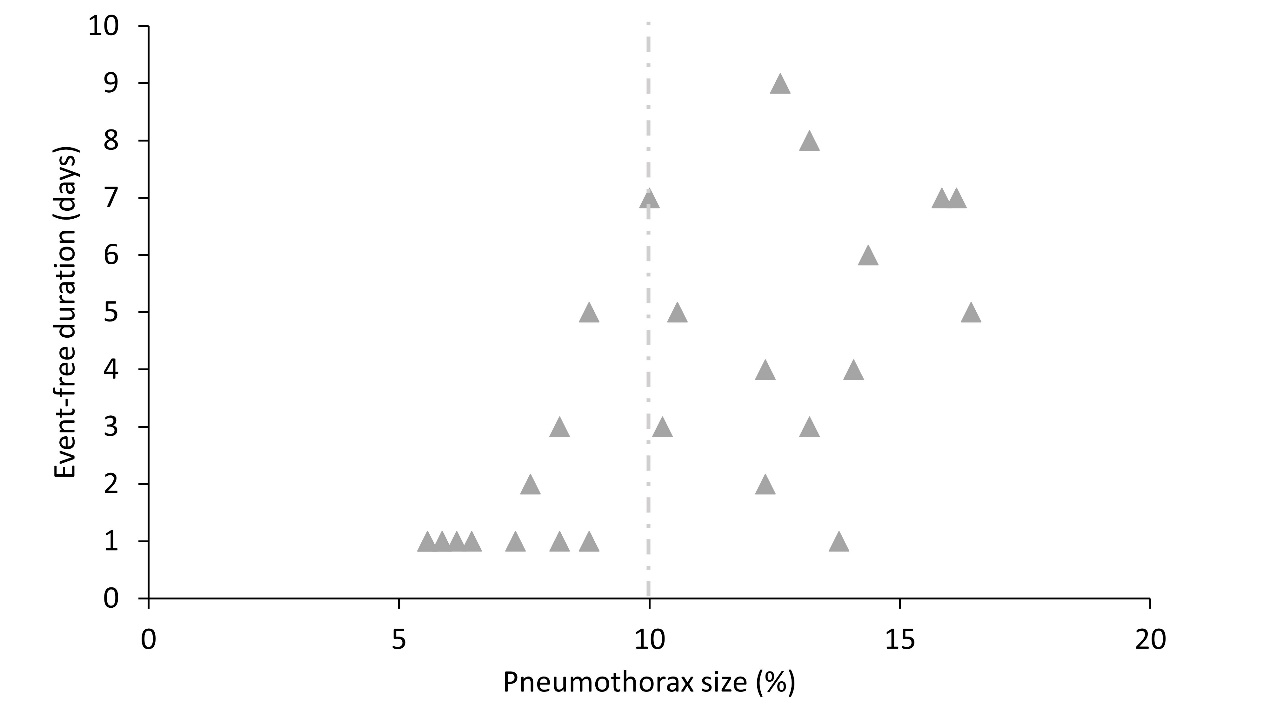
**

*An intercept line at 10% separates patients into two groups with different event-free durations.

**Supplementary Table S1.** Comparison of Studies Employing Simple Air Aspiration in Patients with Iatrogenic or Traumatic Pneumothoraces.

|  | Study design | Enrolment criteria | Method of aspiration | Patient number and success rate*, n (%) | Procedure time and hospital duration | Pain, costs, and complications |
| --- | --- | --- | --- | --- | --- | --- |
| Chen *et al.*, 2022 | Prospective cohort study | Inclusion: Radiographic evidence of pleural line after lung biopsy and the size of pneumothorax ≥ 15% (Rhea’s criteria)  Exclusion:  Age < 20 years, bleeding tendency, and hemodynamic instability | 16G IV catheter, a 3- way stopcock, drainage set, and vacuum bottle | Total patients: 21  Success: 15  (71.4%) | Procedure time:  median 90 (IQR 60-180) seconds  Hospital duration:  Median 2  (IQR 1-4) days | Pain:  - before: median 1 (IQR 0-1)  - during: median 1 (IQR 0-1)  - after: median 0 (IQR 0-1)  Cost:  Vacuum bottle plus catheter aspiration: 49 USD Pigtail drainage: 136 USD  Complications: nil |
| Domokos *et al.*, 2020 [1] | Retrospective cohort study | Inclusion:  Pneumothorax with visible rim ≥ 2 cm between the lung margin and the chest wall at the level of the hilum  Exclusion:  N/A | 16-G or 18-G over the needle cannula, a 3-way stopcock, and 50-mL syringe | Total patients:14  Success: 8  (57.1%) | Hospital duration:  2.0 (IQR 2.0-3.25) days |  |
| Parlak *et al.*, 2012 [2] | Prospective randomized controlled study | Inclusion:  age 18-85 years, first symptomatic pneumothorax or asymptomatic with size ≥ 20% (Light's index)  Exclusion:  Pregnancy, severe comorbidity, recurrent or tension pneumothorax, limited decision-making, chronic lung disease, HIV or Marfan syndrome | 1.3-mm angio intravenous catheter, a 3-way valve, and 50-mL syringe | Total patients: 25  Success: 17  (68%) ** | Hospital duration: 2.4 ± 2.6 days** |  |
| Yamagami *et al.*, 2006 [3] | Prospective cohort study | Inclusion:  Post CT-guided biopsy with pneumothorax not considered to be small (≥7 slices on post-biopsy CT) irrespective of symptoms  Exclusion:  N/A | 18-G IV catheter, a 3-way stopcock, and 50-mL syringe under real-time CT fluoroscopy guidance | Total patients: 72  Success: 61  (84.7%) | Hospital duration:   - Complete resolution: 1.9 ± 2.0 (1 day, 0-7) days - Partial resolved: 4.1 ± 2.4 (3, 0-8) days |  |
| Faruqi *et al.*, 2004 [4] | Prospective cohort study | Inclusion:  Size of pneumothorax ≥15% of hemithorax or symptomatic  Exclusion:  Very sick patient or tension pneumothorax | 18-G IV catheter, a 3-way stopcock, and 50-mL syringe | Total patients: 12  Success: 11  (91.7%) | Hospital duration:   - Aspiration only: 1.6 days - Aspiration failed followed with intercostal tube: 10 days - Direct with intercostal tube: 8.2 days | Pain:  VAS 1.6 (aspiration)  VAS 4.2 (failed with intercostal tube)  VAS 4.0 (intercostal tube)  Cost:  Simple aspiration: 90 IRP  Direct intercostal tube: 300 IRP |
| Yamagami *et al.*, 2002 [5] | Prospective cohort study | Inclusion:  Post CT-guided biopsy with pneumothorax not considered to be small (≥7 slices on post-biopsy CT) irrespective of symptoms  Exclusion:  N/A | 18-G IV catheter, a 3-way stopcock, and 50-mL syringe under real-time CT fluoroscopy guidance | Total patients: 20  Success: 18  (90%) | Hospital duration:  3.60 ± 2.78 (range, 0-9; median, 3) days |  |
| Yankelevitz *et al.*, 1996 [6] | Prospective cohort study | Inclusion:  Post CT-guided biopsy with large size pneumothorax (visually estimated >30% on CT)  Exclusion:  N/A | 18-G 5-cm IV catheter, a 3-way stopcock, and 50-mL syringe | Total patient:17  Success: 12  (70.6%) | Procedure duration: 10-15min | Complication: nil |
| Markos *et al.*, 1990 [7] | Prospective cohort study | Inclusion:  Symptomatic pneumothorax (dyspnea, chest pain) visually ≥ 20% of hemithorax by PA CXR  Exclusion:  Severe respiratory distress, simultaneous bilateral pneumothoraxes, post pneumonectomy | 16-G IV catheter, a 3-way stopcock, and 60-mL syringe | Total patients: 12  Success: 8  (67%) | Hospital duration:  success: 1.13 ± 0.35 days failed: 3.50 ± 0.71 days | Cost: No exact amount but mentioning that the cost of simple aspiration is 1/10th of that of the large intercostal catheter  Complication:  local subcutaneous emphysema (6 patients) and mild vasovagal reaction (2 patients) |
| Delius *et al.*, 1989 [8] | Prospective cohort study | Inclusion:  Age ≥16 years, simple uncomplicated pneumothorax on chest X-ray  Exclusion:  Pleural effusion, hemothorax, multiple traumas, respiratory distress, hemodynamic instability | 8-F radiopaque polytetrafluoroethylene (Teflon) catheter with a 3-way stopcock, and a 50-mL syringe | Total patients:79  Success: 59  (74.7%) | Hospital duration: N/A (Discharge after 6 hours) | Cost:  catheter aspiration: 868 USD Heimlich valve: 2884 USD Heimlich valve plus suction: 3028 USD Chest tube: 6402 USD  Complication:  1 hemothorax; 2 retained sheared catheter tips |
| Talbot *et al.*, 1986 [9] | Prospective cohort study | Inclusion:  Age ≥16 years, simple uncomplicated pneumothorax on chest X-ray  Exclusion:  Cardiopulmonary instability, presence of hemothorax, hydrothorax, complex pulmonary disease | 16-G IV catheter, a 3-way stopcock, and a 50-mL syringe | Total patients: 57  Success: 46  (80.7%) | Hospital duration:  5.8 (4-10) days |  |
| Obeid *et al.*, 1985 [10] | Prospective cohort study | Inclusion:  Simple traumatic pneumothorax  Exclusion:  Hemodynamic unstable; clinically important injuries; hemothorax; hydrothorax; pulmonary disease; respiratory distress | 16-G IV catheter, a 3-way stopcock and a 50-mL syringe | Total patients:17  Success at 1st attempt: 14/17 (82.4%) Success after 2nd attempt: 16/17 (94.1%) | Hospital duration:  N/A (no hospitalization) | Cost:  Aspiration: 310 USD Chest tube: 3,030 USD |

G: gauge, IV: intravenous, N/A: not applicable

*Success was defined as aspiration only without subsequent rescue method such as tube thoracostomy.

**The study did not separate patients with pneumothorax of different etiologies (included both spontaneous pneumothorax and traumatic pneumothorax)

Reference of Table 3:

1. Domokos D, Szabo A, Banhegyi G, Polgar B, Bari Z, Bogyi P, Marczell I, Papp L, Kiss RG, Duray GZ *et al*: **Needle aspiration for treating iatrogenic pneumothorax after cardiac electronic device implantation: a pilot study**. *J Interv Card Electrophysiol* 2020, 57(2):295-301.

2. Parlak M, Uil SM, van den Berg JW: **A prospective, randomised trial of pneumothorax therapy: manual aspiration versus conventional chest tube drainage**. *Respir Med* 2012, 106(11):1600-1605.

3. Yamagami T, Kato T, Hirota T, Yoshimatsu R, Matsumoto T, Nishimura T: **Duration of pneumothorax as a complication of CT-guided lung biopsy**. *Australas Radiol* 2006, 50(5):435-441.

4. Faruqi S, Gupta D, Aggarwal AN, Jindal SK: **Role of simple needle aspiration in the management of pneumothorax**. *Indian J Chest Dis Allied Sci* 2004, 46(3):183-190.

5. Yamagami T, Nakamura T, Iida S, Kato T, Nishimura T: **Management of pneumothorax after percutaneous CT-guided lung biopsy**. *Chest* 2002, 121(4):1159-1164.

6. Yankelevitz DF, Davis SD, Henschke CI: **Aspiration of a large pneumothorax resulting from transthoracic needle biopsy**. *Radiology* 1996, 200(3):695-697.

7. Markos J, McGonigle P, Phillips MJ: **Pneumothorax: treatment by small-lumen catheter aspiration**. *Aust N Z J Med* 1990, 20(6):775-781.

8. Delius RE, Obeid FN, Horst HM, Sorensen VJ, Fath JJ, Bivins BA: **Catheter aspiration for simple pneumothorax. Experience with 114 patients**. *Arch Surg* 1989, 124(7):833-836.

9. Talbot-Stern J, Richardson H, Tomlanovich MC, Obeid F, Nowak RM: **Catheter aspiration for simple pneumothorax**. *J Emerg Med* 1986, 4(6):437-442.

10. Obeid FN, Shapiro MJ, Richardson HH, Horst HM, Bivins BA: **Catheter aspiration for simple pneumothorax (CASP) in the outpatient management of simple traumatic pneumothorax**. *J Trauma* 1985, 25(9):882-886.
